# Biogeography of ammonia oxidizers in New England and Gulf of Mexico salt marshes and the potential importance of comammox

**DOI:** 10.1038/s43705-021-00008-0

**Published:** 2021-03-29

**Authors:** A. E. Bernhard, J. Beltz, A. E. Giblin, B. J. Roberts

**Affiliations:** 1 Department of Biology, Connecticut College, New London, CT USA; 2 Ecosystems Center, Marine Biological Laboratory, Woods Hole, MA USA; 3 Louisiana Universities Marine Consortium, Chauvin, LA USA; 4 Present Address: School of Arts and Sciences, University of Pennsylvania, Philadelphia, PA USA

**Keywords:** Microbial ecology, Biogeochemistry

## Abstract

Few studies have focused on broad scale biogeographic patterns of ammonia oxidizers in coastal systems, yet understanding the processes that govern them is paramount to understanding the mechanisms that drive biodiversity, and ultimately impact ecosystem processes. Here we present a meta-analysis of 16 years of data of ammonia oxidizer abundance, diversity, and activity in New England (NE) salt marshes and 5 years of data from marshes in the Gulf of Mexico (GoM). Potential nitrification rates were more than 80x higher in GoM compared to NE marshes. However, nitrifier abundances varied between regions, with ammonia-oxidizing archaea (AOA) and comammox bacteria significantly greater in GoM, while ammonia-oxidizing bacteria (AOB) were more than 20x higher in NE than GoM. Total bacterial 16S rRNA genes were also significantly greater in GoM marshes. Correlation analyses of rates and abundance suggest that AOA and comammox are more important in GoM marshes, whereas AOB are more important in NE marshes. Furthermore, ratios of nitrifiers to total bacteria in NE were as much as 80x higher than in the GoM, suggesting differences in the relative importance of nitrifiers between these systems. Communities of AOA and AOB were also significantly different between the two regions, based on *amo*A sequences and DNA fingerprints (terminal restriction fragment length polymorphism). Differences in rates and abundances may be due to differences in salinity, temperature, and N loading between the regions, and suggest significantly different N cycling dynamics in GoM and NE marshes that are likely driven by strong environmental differences between the regions.

## Introduction

Nitrification in salt marshes plays a critical role in the fate of nitrogen, yet we lack a full understanding of the distribution and diversity of nitrifiers in geographically distinct marshes, and how these differences may impact ecosystem processes. Two of the major groups of microorganisms carrying out nitrification, ammonia-oxidizing archaea (AOA) and bacteria (AOB), almost always coexist in coastal habitats, but how these two groups partition resources and contribute to nitrification under different conditions has yet to be resolved.^1,2^ Although previous studies of nitrifier biogeography have suggested that dominant taxa tend to be globally distributed,^3^ the patterns observed are not always explained by the environmental variables analyzed,^4^ leaving questions about the forces driving their biogeography. The recent discovery of a complete nitrifier, comammox,^5^ further complicates our understanding of niche partitioning among nitrifying microorganisms.

In a recent comparison of nitrification rates among disparate marshes and estuarine systems, Marton et al.^6^ reported differences of more than three orders of magnitude in some cases, with rates in Gulf of Mexico (GoM) marshes far surpassing those reported in other marshes. Some of the variation may be due to differences in time of year, vegetation, or sediment chemistry, but it may also be a reflection of differences in nitrifiers present. There is evidence that there may be cosmopolitan phylotypes found in similar systems, but there have been few direct comparisons between geographically distant marsh systems (although see^3^ for AOB). Ratios of abundances of AOA and AOB can also vary by orders of magnitude in estuarine systems, and, unlike open ocean systems where AOA always outnumber AOB, neither group has emerged as a clear dominant in estuarine systems.^2^ Based on studies of cultivated isolates, nitrifiers have different physiological profiles, with greater affinity for ammonium found among AOA^7^ and comammox,^8^ providing strong mechanisms for niche partitioning with AOB. Additionally, AOB are known to contribute more N_2_O to the atmosphere during nitrification relative to AOA.^9^ Understanding distribution patterns of nitrifiers, and how these relate to process rates, can lead to a better understanding of the fate of nitrogen and its impact on the environment.

Given the high nitrification rates previously reported in the GoM compared to more northern Atlantic marshes,^6^ we hypothesized that geographic differences in ammonia oxidizer communities might explain the differences in rates. Studies of nitrifier communities have been conducted in Atlantic coastal systems, including New England (NE)^10–12^ and the Chesapeake Bay,^13,14^ and in the GoM,^15–18^ but no cross-region comparisons have been made. If significant community differences occur, it would suggest that biogeographical patterns of ammonia oxidizers may translate into differences in N processing in the GoM compared to other marshes, where rates are more moderate.

In this study, we took advantage of data collected over 16 years from salt marshes in NE and over 5 years in marshes in the GoM. We initially set out to answer the following questions: (1) Does the abundance and composition of AOA and AOB differ significantly among geographically distant marshes that differ significantly in nitrification rates? (2) What are the factors that drive spatial variability of AOA and AOB? and (3) Are there common factors that regulate AOA and AOB abundance and diversity in salt marshes? With the recent discovery of the complete nitrifier, comammox, we added a fourth question: Are comammox bacteria potentially an important player in these marshes? Answers to these questions should help us to understand how abundance and diversity of ammonia oxidizers influence nitrification rates in salt marshes, and how changes in environmental conditions might impact N cycling in coastal systems more broadly.

## Materials and methods

### Site descriptions

**Fig. 1:**
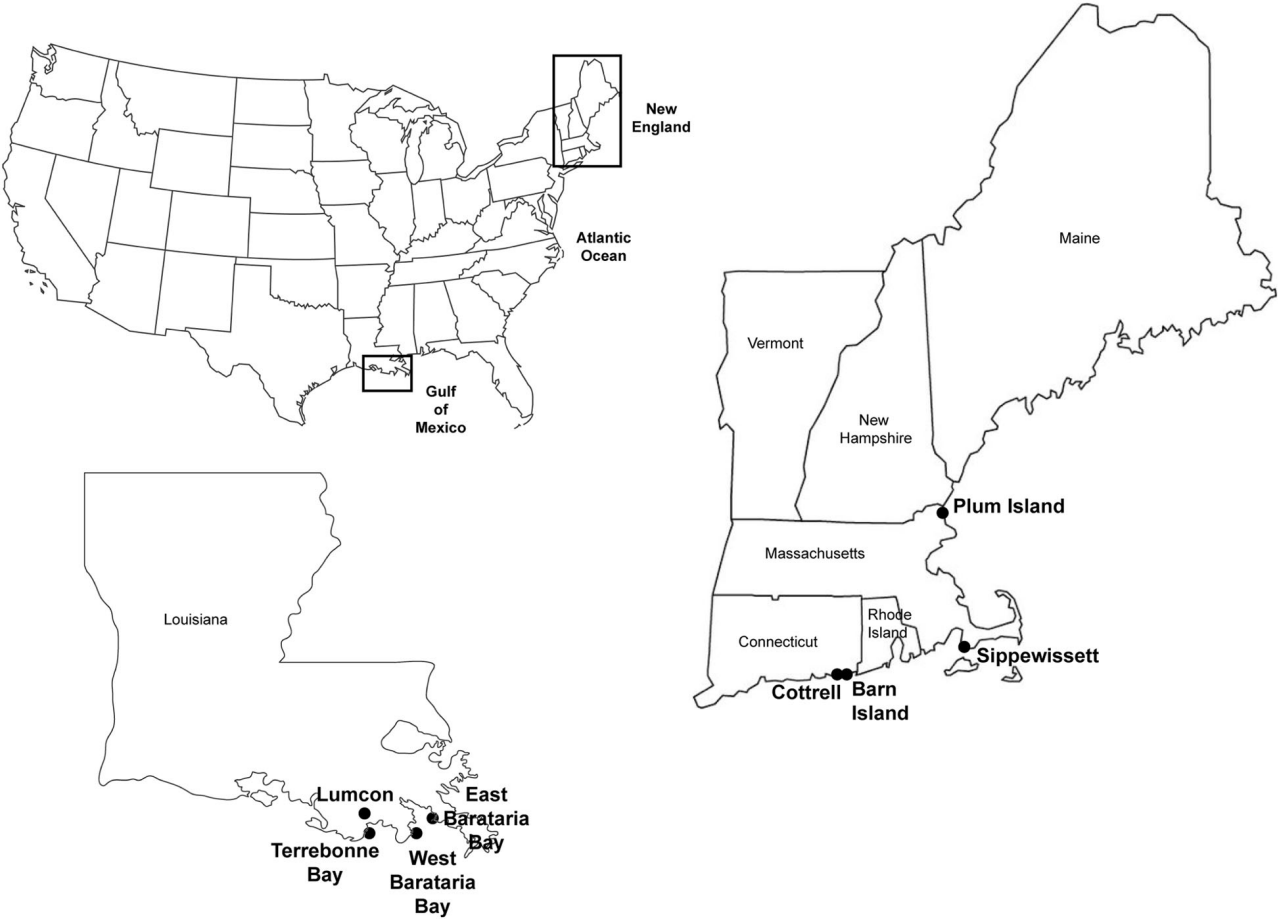
Location of marshes in the Gulf of Mexico and New England sampled in this study. Sampling sites within each region are indicated in the enlarged portions of the map.

Samples used in this study were collected from eight different marsh areas, four in NE and four in the GoM (Fig. 1). Samples from NE were collected from Barn Island (BI) and Cottrell (CO) marshes in southeastern Connecticut, the Great Sippewisset Marsh (GSM) on Cape Cod, and the Plum Island Estuary (PIE) in northeastern Massachusetts on 18 different sampling dates from April 2001 to July 2017 (Table S1). At all sites, only control (unfertilized) sites were included in the analyses. Sampling procedures and site descriptions have been previously published.^10,19–21^ Dominant vegetation in NE marshes varied, with most samples collected in plots dominated by *Spartina patens* or *Spartina alterniflora* (tall form), but others were dominated by short *S. alterniflora*, *Juncus gerardii, Distichlis spicata*, forbs, mixed vegetation, and unvegetated sediments.

Sediment samples from GoM marshes were collected at a total of 15 marsh sites in Terrebonne Bay (TB), western and eastern Barataria Bay (WB and EB, respectively) as well as near the LUMCON DeFelice Marine Center in Cocodrie, LA (LUM). These sites, spanning ~100 km of the Louisiana Gulf Coast, were sampled on 14 different sampling dates from May 2012 to July 2016 (Table S1). Detailed site descriptions are found elsewhere.^6,18,22,23^ The GoM marshes in this study were all dominated by *S. alterniflora*, although all sites also contained some combination of *Juncus roemarianus*, *Distichlis spicata*, *S. patens*, and/or *Avicennia germinans*. All sampling took place within plots dominated by *S. alterniflora* except for a small number of samples collected from *J. romerianus* or unvegetated areas at LUM.

### Sample collection and processing

Most of the samples from NE marshes represent the top 2 cm of sediment, however, we also collected sediments down to 4 cm in some cases in BI, PIE, and GSM marshes. DNA was extracted using the MoBio PowerSoil DNA kit (MoBio, Carlsbad, CA) or the DNeasy Powersoil kit (Qiagen, Germantown, MD). Potential nitrification rates were measured as previously described.^11,24^ Methods for sediment and porewater (pw) properties in NE marshes (soil moisture, pw salinity, pw pH, pw NH_4_^+^) have been previously published.^10,11,20^ Water temperatures were obtained for Long Island Sound (longislandsoundstudy.net), Plum Island Sound (ecosystems.mbl.edu/PIE/data-archive/EST/EST-PR-O2.html), and Cape Cod (seatemperature.org/north-america/united-states/woods-hole.html) to compare with bay water temperatures in the GoM (Table 1).

**Table 1:** Mean (SE) values for potential nitrification rates (PNR) and sediment properties for GoM and NE marshes.

Region	Marsh	PNR (µM-N/g/day)	Salinity (psu)	NH_4_^+^ (µM-N)	% Water	pH	Water temp (°C)
GoM	All marshes combined	712.9 (57.1)	13.6 (0.4)	88.3 (12.0)	73.0 (0.4)	6.9 (0.04)	31.9 (0.3)
	EB	901.5 (130.7)	9.2 (0.4)	54.8 (7.4)	74.7 (0.5)	6.7 (0.06)	32.2 (0.4)
	TB	526.9 (85.0)	15.2 (0.5)	147.0 (27.6)	77.0 (0.4)	7.2 (0.05)	32.6 (0.4)
	WB	1027.2 (115.0)	20.4 (1.0)	52.4 (10.9)	65.5 (0.9)	6.8 (0.06)	29.9 (0.5)
	LUM	77.7 (16.4)	10.0 (0.3)	nd	75.9 (0.4)	7.7 (0.03)	29.4 (0.17)^a^
NE	All marshes combined	30.1 (5.4)	21.1 (0.8)	88.9 (10.8)	61.7 (1.0)	6.1 (0.05)	21.6 (0.2)^b^
	BI	16.7 (8.6)	24.7 (0.9)	73.6 (12.5)	63.4 (1.7)	6.0 (0.05)	20.1 (0.1)^b^
	CO	nd	26.6 (1.4)	nd	64.0 (2.6)	6.2 (0.09)	20.1 (0.1)^b^
	PIE	35.3 (6.6)	13.7 (1.3)	156.4 (21.9)	47.5 (1.7)	nd	21.8 (2.7)^b^
	SIP	nd	23.5 (1.6)	25.0 (2.8)	79.9 (1.1)	nd	19

^a^Average soil temperature at sample sites (no water temp data available).

^b^Temperatures were obtained from the following sites for Niantic Bay in Long Island Sound (longislandsoundstudy.net), Plum Island Sound (ecosystems.mbl.edu/PIE/data-archive/EST/EST-PR-O2.html), and Cape Cod (seatemperature.org/north-america/united-states/woods-hole.html).

For all GoM marshes, sample collection (the top 5 cm) and potential nitrification rates were performed according to Marton et al.^6^ and Schutte et al.^25^. Methods for sediment and pw properties (pw salinity, NH_4_^+^, and soil moisture) have also been previously published.^6,22^ Sediment for DNA analysis was stored at −80 °C until extracted, using the MoBio PowerSoil DNA kit (MoBio) or the DNeasy Powersoil kit (Qiagen).

### Gene abundances

Much of the data for AOA and AOB have been previously published,^6,11,17,18,20,24,26^ but we included additional samples to obtain a more complete data set. Bacterial 16S rRNA, archaeal *amo*A, and betaproteobacterial *amo*A genes were quantified using previously published primers and protocols (Table S2).

Comammox clade A *amo*A genes were amplified using the complete primer mix described in Pjevac et al.^27^, but we were unsuccessful in obtaining specific products in most samples, likely due to the high number of degeneracies in the mix. Therefore, we tested each possible combination of forward and reverse primers to identify which primers produced specific product. We then used only those primers that gave a specific product based on visualization of the products in a 1% agarose gel stained with Gel Red under UV light. Only one forward primer (comaA-244f_d) and two reverse primers (comaA-659r_c and comaA-659r_d) produced the desired product, so we used an equimolar mix of the two reverse primers with the single forward primer for QPCR and sequence analysis. We also tested samples for the presence of comammox clade B with the primers from Pjevac et al.^27^ using the same approach as with the clade A primers (testing all combinations), but were unsuccessful in amplifying the correct-sized product.

All samples were run in duplicate for QPCR analysis. Optimal dilutions for each set of samples were empirically determined by running multiple dilutions and calculating the slopes. Based on these empirical data, samples from the GoM sites were run undiluted for the final analysis, while samples from NE sites were run diluted 10X. Conditions are found in the Supplementary Materials (Table S2).

Archaeal *amo*A genes were quantified using either CrenAmoAQ-ModF^11^ and ArchAmoAR^28^ or ArchAmoA26F and ArchAmoA417R.^29^ Our archaeal *amo*A data set is composed of samples collected from different marshes in different years for different studies. For each study, different primer sets were tested initially on a subset of samples, and the archaeal *amo*A primer set generating the most consistent and robust data was used to analyze all samples collected for that study. Coincidentally, archaeal *amo*A genes from the GoM marshes were amplified using ArchAmoA26F/417R, while archaeal *amo*A genes from NE marshes were amplified using primers CrenAmoAQ-ModF/ArchAmoAR. To ensure that the differences in archaeal *amo*A gene abundances were not simply due to primer bias, we ran subsets of GoM and NE samples with both primer sets for comparison. The subset for GoM consisted of 97 samples, representing TB, EB, and WB from May, July, August, and September, and abundances spanned the range of abundances in the full data set. For GoM samples, there was no difference in archaeal *amo*A amplification between primer sets (*r*^2^ = 0.90, slope = 1.01, *n* = 97). The subset for NE consisted of 115 samples, representing all 4 marshes, 2 depths, and all vegetation types collected in April, July, September, and October, and spanned the range of abundances in the full data set. We found significantly lower gene abundances for NE samples amplified with the ArchAmoA26F/417R primers compared to CrenAmoAQ-ModF/ArchAmoAR primers and no correlation between the two data sets (*r*^2^ = 0.002, slope = 0.02, *n* = 115). Since archaeal *amo*A abundances determined with CrenAmoAQ-ModF/ArchAmoAR primers from an NE marsh have been previously confirmed by comparison to 16S rRNA gene amplification,^13^ we chose to use archaeal abundances generated with different primer sets in the two regions to best represent archaeal *amo*A abundances.

### Terminal restriction fragment length polymorphism (TRFLP) profiles

Community DNA profiles of archaeal and betaproteobacterial *amo*A genes were generated by TRFLP analysis as previously described.^17,26^ Since previous archaeal *amo*A TRFLP of NE samples had been done using different primers, we repeated the analysis using Arch26F/417R, so that samples in both regions could be compared directly. Although the Arch26F/417R primer pair has mismatches with some sequences detected in NE marshes, we felt this approach was better than comparing communities using different primers. We did not conduct TRFLP analysis for comammox genes.

### Sequence analysis

Archaeal and betaproteobacterial *amo*A sequences were obtained from previously published studies.^10,11,17,20^ We also generated new archaeal and betaproteobacterial *amo*A sequences from GoM samples collected in 2016, and comammox genes from both GoM and NE samples, for a total of 1119 archaeal, 890 betaproteobacterial, and 98 comammox *amo*A sequences in our final analyses (Table S1). Archaeal and betaproteobacterial *amo*A genes were cloned and sequenced according to the protocol in Bernhard et al.^17^. Comammox clade A *amo*A genes were amplified using comaA-244f_d paired with equimolar mixture of comaA-659r_c and comaA-659r_d and cloned using Strataclone PCR cloning kit (Agilent, Santa Clara, CA) following the manufacturer’s directions. Sequences were deposited into the GenBank database under accession numbers MK487157-MK487377 and MW022959-MW023057. AOA and AOB sequences were aligned in ARB^30^ and manually checked. Comammox sequences were aligned in MEGA v.7.^31^ Phylogenetic trees were constructed using the neighbor-joining algorithm in either ARB or MEGA. Bootstrap analysis was conducted for all *amo*A analyses in MEGA. OTUs were identified using the cluster function in mothur^32^ with a 95% similarity cutoff.

### Statistical analyses

All data were tested for normality by Shapiro–Wilkes tests or Q–Q plots using R,^33^ and data were log-transformed when the criteria were not met or, in cases where transformed data still did not meet the criteria, nonparametric tests were used. Differences in abundance among marshes and salinity categories were analyzed by ANOVA in R, using the Tukey’s LSD post hoc test for pairwise comparisons. Linear multiple regressions and Pearson’s correlation analyses were conducted in R. TRFLP data were analyzed using nonmetric multidimensional scaling (NMDS) as previously described.^20^ Diversity indices, richness and Simpson’s inverse (1/D′), were calculated from TRFLP and sequence data (for AOA and AOB), using number of TRFs and number of OTUs as the unit of diversity.

## Results

### Potential nitrification rates

**Fig. 2:**
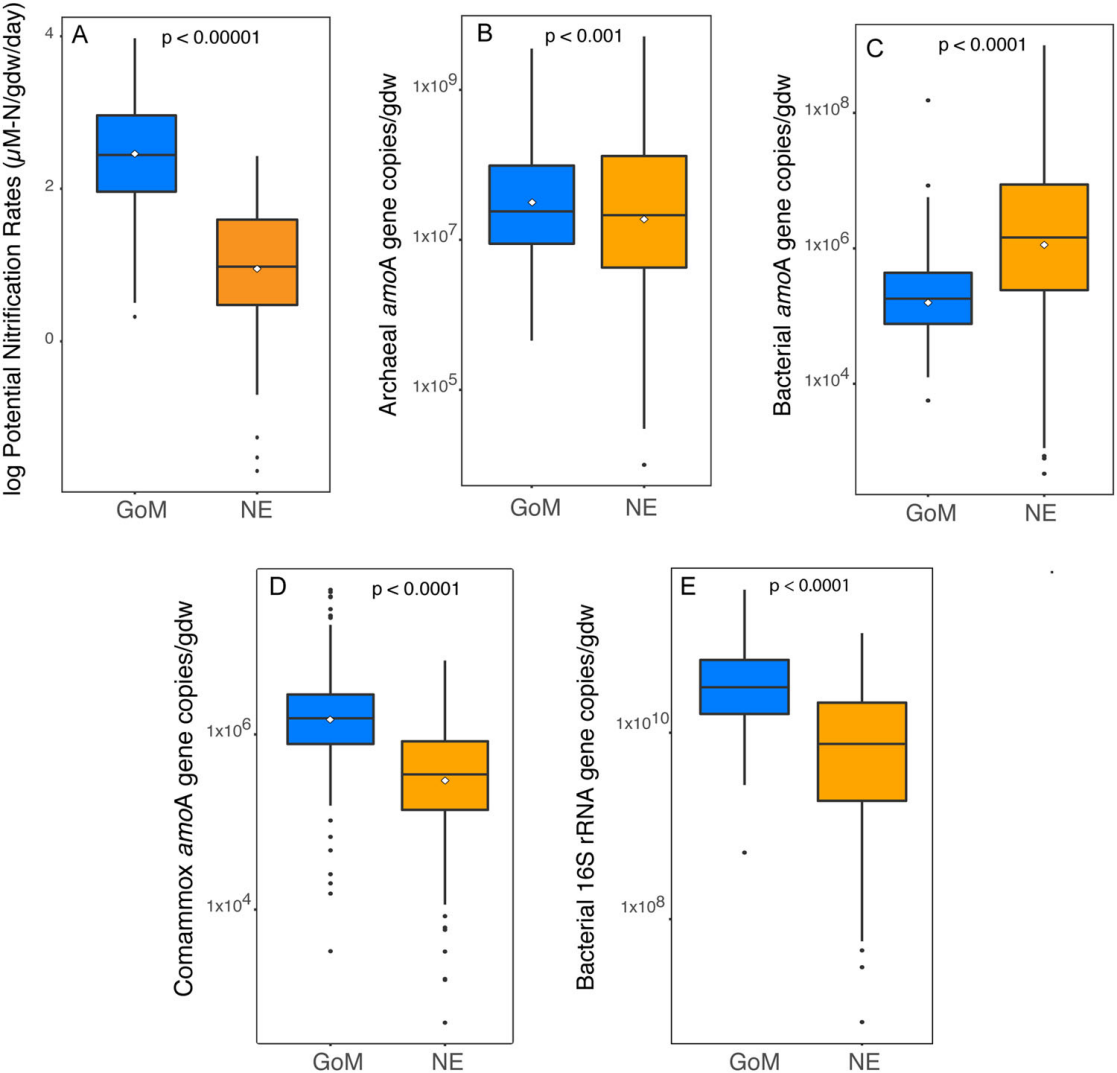
Gene abundances and potential nitrification rates in GoM and NE marshes. Potential nitrification rates (panel (**A**)), and abundance of archaeal *amo*A (panel (**B**)), betaproteobacterial *amo*A (panel (**C**)), comammox (panel (**D**)) *amo*A, and Bacterial 16S rRNA (panel **E**) genes in marshes in the Gulf of Mexico (blue boxes) and New England (orange boxes). White diamonds represent the means. Significance values from Wilcoxon-rank tests comparing ratios between the two regions are indicated in each panel.

Rates were analyzed from a total of 577 samples (463 from GoM marshes and 114 from NE marshes). Rates in GoM marshes were more than 50 times higher on average compared to rates in NE marshes (Fig. 2A). Rates also varied among the marshes in the GoM region, with rates at EB and WB significantly higher (*P* < 0.0001) than TB and LUM (Fig. S1A). Rates within the NE marshes where rates were measured (BI and PIE) were not significantly different from each other.

### AOA abundance

We analyzed abundance of AOA from 760 samples (527 from GoM marshes, 233 from NE marshes). Abundance of AOA varied by more than four orders of magnitude among the eight marshes (Fig. S1B), and was not consistently higher in one region, but was overall significantly higher in GoM marshes compared to NE marshes (Fig. 2B). Within GoM, AOA abundance was significantly higher in EB compared to the other three marshes (ANOVA, *P* < 0.00001, *F* = 25.6), but no differences in AOA abundance were detected among the other three GoM marshes or among NE marshes (Fig. S1B).

Because all of the GoM samples were collected from the top 5 cm of sediment, and NE marshes were primarily collected from the top 2 cm, we wanted to test whether differences in abundance were simply due to differences in sediment depth. Since we had samples from the 2 to 4 cm depth from some of the NE marshes, we created a subset of data to compare abundances from 0–5 cm in the GoM marshes to 0–4 cm in the NE marshes, so that the difference in depth was minimized. Comparing samples from GoM (0–5 cm) and 0–4 cm samples from NE (representing BI, PIE, and GSM marshes), AOA abundance in GoM marshes was still significantly higher than in NE marshes (*P* = 0.046). We also detected no difference in AOA abundance when comparing 0–2 cm with 2–4 cm samples in NE marshes (*P* = 0.89).

### AOB abundance

We analyzed AOB abundance from 770 samples (545 from GoM marshes, 225 from NE marshes). Abundance of AOB was 22 times greater in NE marshes compared to GoM marshes (1.6 × 10^7^ vs 7.2 × 10^5^ copies/gdw), and the difference was significant (*P* < 0.0001) (Fig. 2C). Comparing abundances in 0–5 cm samples in GoM to 0–4 cm samples in NE, AOB were still significantly greater in the 0–4 cm NE samples compared to the 0–5 cm GoM samples (*P* < 0.0001). When individual marshes were analyzed separately, AOB abundances in all four NE marshes were significantly higher than all four GoM marshes (*P* < 0.0001, *F* = 20.1), and there were no differences among the four NE marshes (*P* = 0.2) (Fig. S1C). In GoM marshes, AOB abundance differed significantly among marshes (*P* < 0.0001, *F* = 11.3).

### Comammox abundance

We analyzed abundance of comammox clade A from 543 samples (309 from GoM and 211 from NE). Because comammox was not discovered at the time of the initial sampling and analyses, our data set for comammox is somewhat smaller compared to AOA and AOB due to limitations in resources. Clade A comammox bacteria in GoM samples were four times higher compared to NE marshes (Fig. 2D), and comammox bacteria were more abundant than AOB in GoM marshes. Comammox abundance in samples from 0 to 5 cm in GoM was still significantly greater than samples from 0 to 4 cm in NE marshes (*P* < 0.00001). We were unsuccessful in our attempts to detect comammox clade B genes from either region.

### Abundance of total bacteria

We analyzed abundances of total bacterial 16S rRNA genes from 455 samples (279 from GoM and 176 from NE). Bacterial abundance was twice as high in GoM marshes compared to NE marshes (*P* < 0.0001) (Fig. 2E).

### Ratios of nitrifier groups

**Fig. 3:**
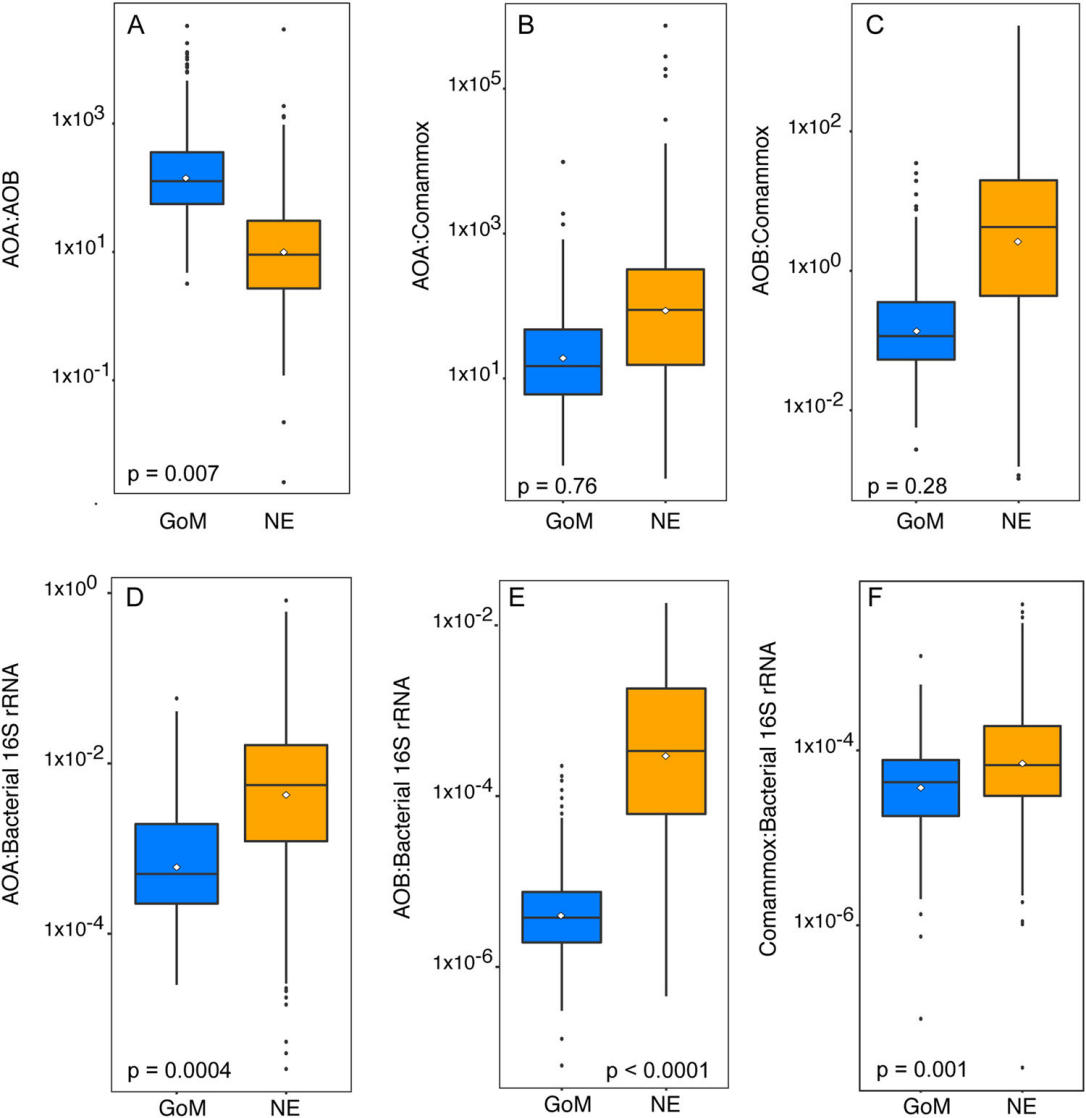
Ratios of the different genes in GoM and NE marshes. Ratios of AOA (panel **A**), AOB  (panel **B**), and comammox clade A (panel **C**) *amo*A genes and ratios of the three *amo*A genes to bacterial 16S rRNA genes (panels (**D**–**F**)) from Gulf of Mexico (blue boxes) and New England (orange boxes) marshes. White diamonds represent the means. Significance values from Wilcoxon-rank tests comparing ratios between the two regions are indicated in each panel.

Ratios of AOA to AOB were significantly higher in GoM marshes compared to NE marshes (Fig. 3A). Ratios were always > 1 in GoM marshes, while in NE marshes, the average ratio was >1, but AOB outnumbered AOA in 14% of the samples. Ratios of AOA to comammox and AOB to comammox were lower in GoM marshes relative to NE marshes, but the differences were not significant (Fig. 3B, C).

Because overall microbial populations in the GoM were greater based on 16S rRNA genes, we compared the abundance of nitrifier to the total bacterial population. Ratios of AOA, AOB, and comammox to total bacterial 16S rRNA genes were ~10x, ~80x, and ~4x higher, respectively, in NE marshes than in GoM marshes, and were significantly different between the two regions (Fig. 3D–F).

### Correlation of abundances and rates

When all samples were combined in a linear multiple regression analysis, potential nitrification rates were significantly positively correlated with AOA (*P* < 0.0001) and AOB (*P* < 0.04), but not comammox (*P* = 0.75). In GoM marshes only, rates were positively correlated with AOA (*P* < 0.0001), AOB (*P* = 0.03), and comammox (*P* = 0.004). Conversely, in NE marshes, potential rates were positively correlated with AOB abundance only (*P* < 0.0001).

AOA and AOB abundances were positively correlated in both regions (Fig. S2). Comammox abundance was positively correlated with both AOA and AOB abundance in GoM marshes, but not in NE marshes.

### Sediment properties

In addition to differences in microbial abundances and nitrification rates, GoM and NE marshes differed significantly in sediment properties. GoM marshes had significantly higher water content (73.0% vs 61.7% in GoM and NE, respectively) and water temperatures (31.9 °C vs 21.9 °C in GoM and NE, respectively) compared to NE marshes (*P* < 0.0001, for both parameters) (Table 1). However, salinity in NE marshes was significantly higher (*P* < 0.0001) than in GoM marshes (21.1 vs 13.6 psu in NE and GoM, respectively). Interestingly, pw ammonium did not differ between the two regions (*P* = 0.87), but pH was significantly higher in GoM marshes (*P* < 0.0001).

### Environmental correlations

**Fig. 4:**
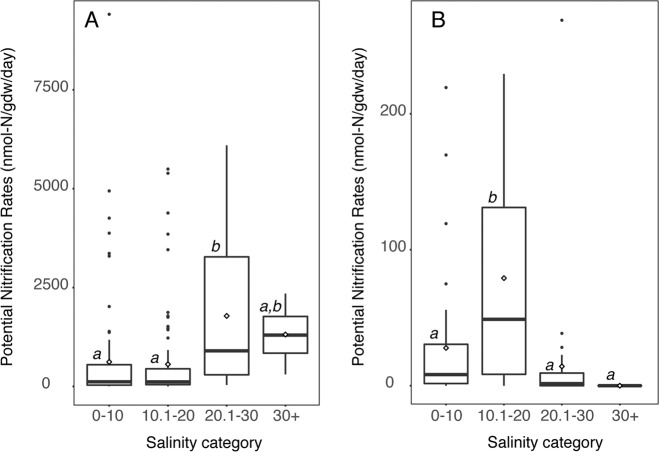
Potential nitrification rates at different salinities in GoM and NE marshes. Rates for GoM (panel **A**) and NE (panel **B**) marshes that are significantly different (*P* < 0.05) between categories are indicated by different letters above the boxes. White diamonds represent mean rates within each category.

In GoM marshes, potential rates were positively correlated with salinity, while in NE marshes, the correlation was negative (Table 2). In the GoM, the maximum average rates were found between 20.1 and 30 psu, while in NE marshes, maximum average rates were between 10.1 and 20 psu (Fig. 4). Additionally, AOB abundance was positively correlated with salinity when data from both regions were combined (*P* = 0.006), but there was no correlation for each region separately (Table 2). Similar to the relationship between rates and salinity, gene abundances for all three groups of nitrifiers showed nonlinear patterns across a salinity gradient, with significant differences with salinity detected for only AOA and AOB in NE marshes and no groups in GoM (Fig. S3). Generally, abundances tended to be highest in the mid-salinity range, except for comammox in NE marshes where the highest abundances were found at salinities over 30.

**Table 2:** Correlation coefficients of potential nitrification rates (PNR) and nitrifier abundance with sediment chemistry.

	Region	% water	Salinity	pH	pw NH_4_^+^
PNR	GoM		0.17		
	NE		(0.22)		
AOA	GoM	0.14*		(0.25)**	
	NE	0.17		(0.41)*	(0.29)
AOB	GoM				
	NE	0.13			
Com	GoM	0.33**		(0.18)	
	NE	0.17			

Only those that were significant (*P* < 0.05) are shown.

*P < 0.01; **P < 0.0001.

In both regions, AOA abundance was correlated with soil moisture and pH, while in NE marshes, AOA abundance was also correlated with NH_4_^+^ (Table 2). AOB abundance in NE marshes was correlated with soil moisture, but interestingly, there were no significant correlations of AOB abundance and sediment properties in GoM marshes.

### AOA community composition

**Fig. 5:**
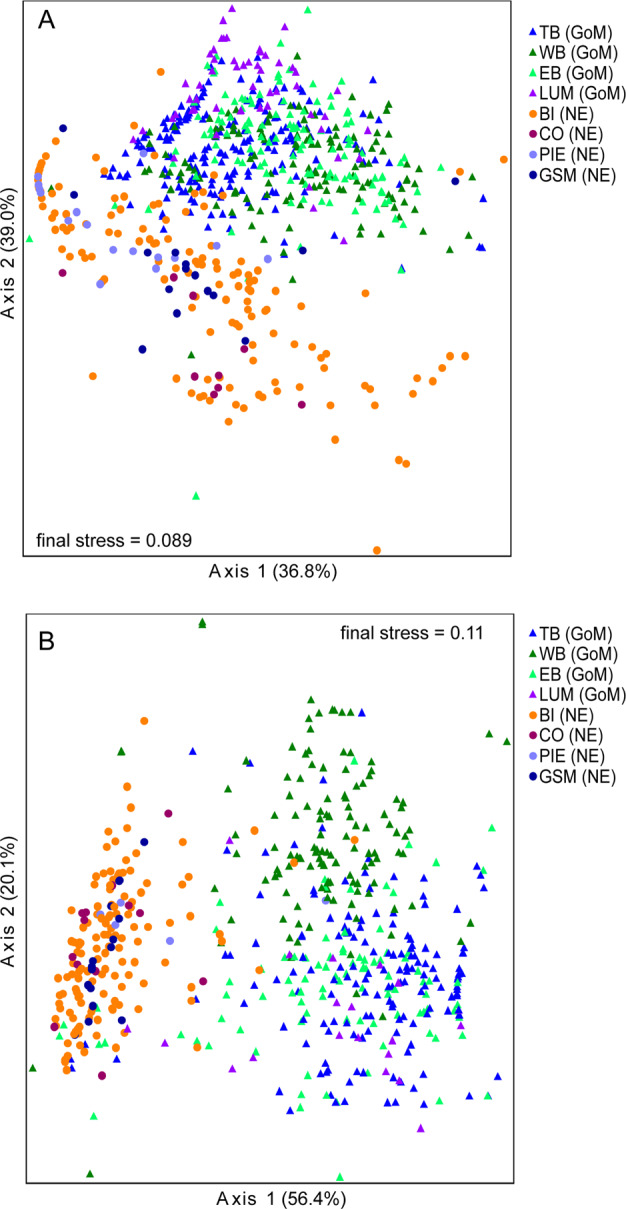
Community composition of AOA and AOB in GoM and NE marshes. Differences in communities were analyzed by nonmetric dimensional scaling for AOA (panel (**A**)) and AOB (panel (**B**)) based on TRFLP analysis of *amo*A genes. Communities from Gulf of Mexico (GoM) marshes are shown by circles, and communities from New England (NE) marshes are shown by triangles. Percent variability explained by each axis of the ordination is indicated parenthetically. Differences between GoM and NE communities were significant (*P* < 0.0001) for both genes.

TRFLP analysis of 677 samples (494 from GoM marshes and 183 from NE marshes) revealed that all eight marshes were dominated by TRF170 (Fig. S4A), which represents *amo*A genes related to *Nitrosopumilus maritimus.*^20^ TRF296 was also abundant in all eight marshes, and also represents sequences related to *N. maritimus*. Six of the nine TRFs were significantly more abundant in NE marshes, and two were significantly more abundant in GoM marshes. NMDS ordination analysis explained over 80% of the variability in the data (Fig. 5A), with significantly different communities in the two regions (MRPP, *P* < 0.00001). The three TRFs most strongly correlated with the separation between the two regions (i.e., axis 1) were TRF83, 170, and 208 (*r* = −0.911, 0.578, and 0.533, respectively). Salinity and pw NH_4_^+^ were positively correlated with ordination axis 1 (*r* = 0.36 and 0.18, respectively), while soil moisture was negatively correlated with axis 1 (*r* = −0.25). Additionally, when samples from both regions were combined and analyzed with salinity parsed into categories (0–10, 10.1–20, 20.1–30, >30), communities were significantly different between categories (*P* < 0.00001), and the pattern was also significant when each region was analyzed separately.

**Fig. 6:**
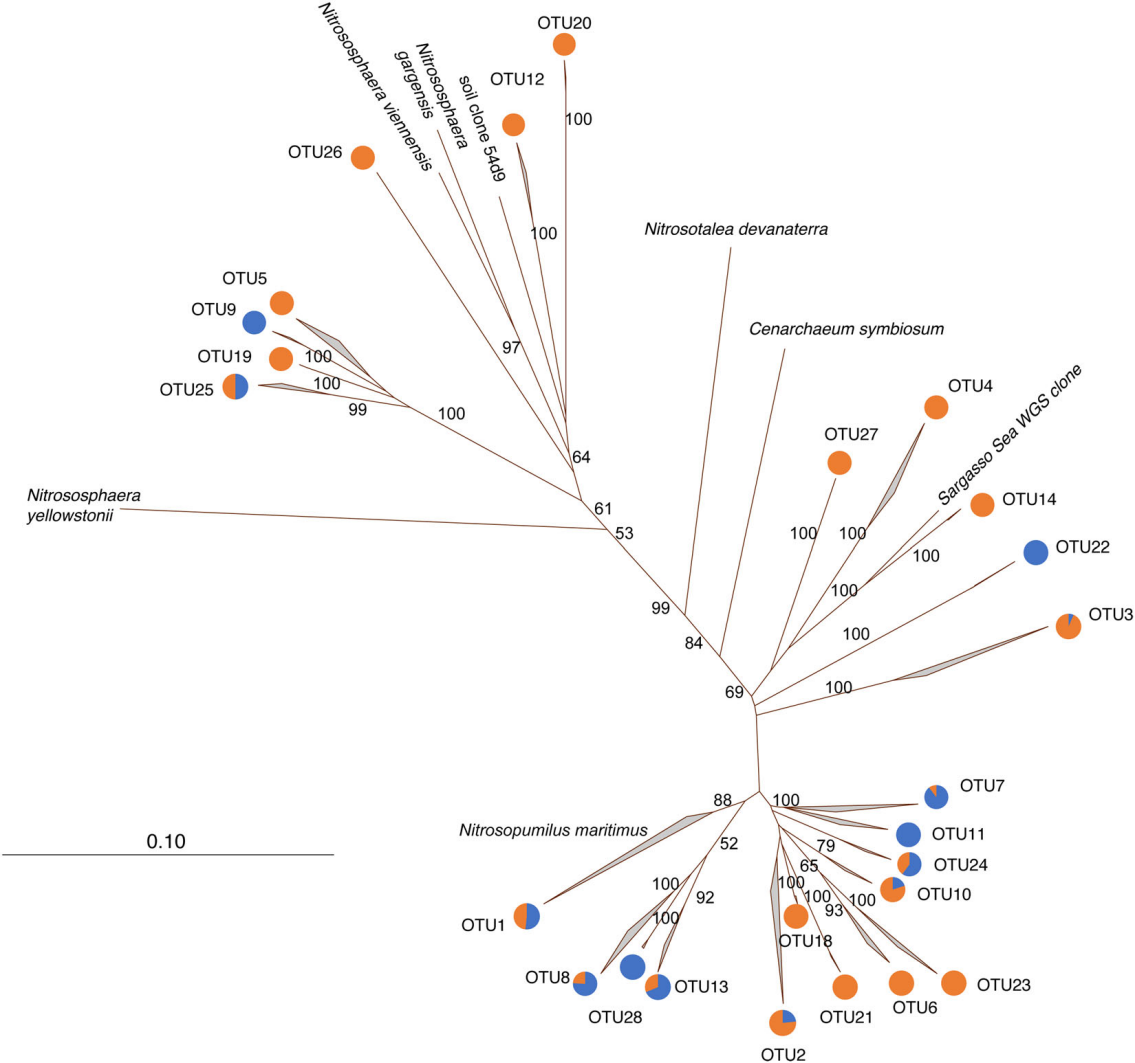
Phylogenetic relationships among archaeal *amo*A OTUs from Gulf of Mexico and New England marshes. The relative abundance of sequences in each OTU from each region is indicated by the pie charts (blue—GoM, orange—NE). Bootstrap values > 50 are indicated on each branch. The scale bar presents number of substitutions per site.

A total of 1119 archaeal *amo*A sequences were included in the analysis (603 from NE marshes and 516 from GoM marshes) (Fig. 6). No sequences from LUM or CO marshes were available, so the sequence data represent three marshes from within each region. A total of 28 OTUs (excluding singletons) were detected at 95% similarity, with 6 of these shared between the two regions. NE marshes had a total of 19 OTUs, while GoM marshes had a total of 14 OTUs. Thirteen OTUs were unique to NE marshes, and 8 were unique to GoM marshes. Furthermore, OTUs 4 and 5 were diagnostic of NE marshes, being found in all three NE marshes, but not in any GoM marshes. Similarly, OTUs 7 and 8 were diagnostic of GoM marshes. OTU1, which includes *N. maritimus*, was the largest, representing 46.7% of all the sequences, and was the only OTU found in all six marshes.

### AOB community composition

TRFLP analysis of 576 samples (411 from the GoM and 165 from NE marshes) shows distinctly different AOB composition in the two regions (Fig. S4B). In the GoM marshes, TRF196 and 336 dominated, while in NE marshes, TRF127 and 130 dominated. TRF278 was the only TRF found in all eight marshes, and represents *Nitrosospira*-like *amo*A sequences. TRF278 also shows a strong negative correlation with axis 2 in the ordination (*r* = −0.83), but the pattern is not explained by any of the environmental variables we measured. In the NMDS ordination, AOB communities formed distinct clusters for each region (Fig. 5B), which were significantly different (MRPP, *P* < 0.00001). Salinity was the factor most strongly correlated with axis 1 (*r* = 0.64). Soil moisture and pw NH_4_^+^ were also correlated with axis 1 (*r* = −0.374 and −0.194, respectively). TRFs 127, 130, and 336 were also strongly correlated with axis 1 in the AOB ordination, and are the strongest drivers of the differences in AOB communities between GoM and NE marshes. Similar to AOA, when samples were analyzed with salinity parsed into categories (0–10, 10.1–20, 20.1–30, >30), AOB communities in the four categories were significantly different (*P* < 0.00001), and the pattern was also significant when samples from within each region were analyzed separately.

**Fig. 7:**
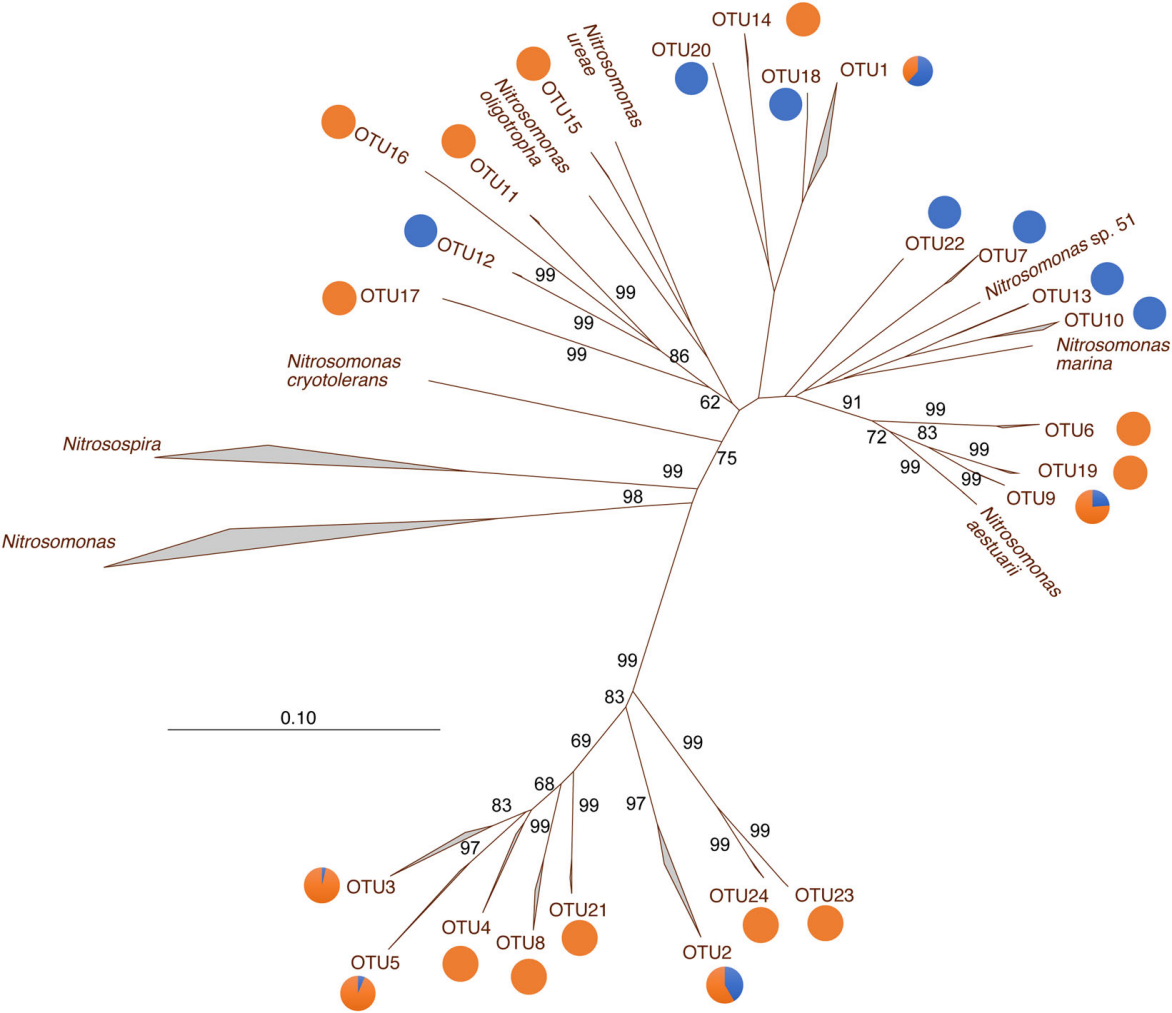
Phylogenetic relationships among betaproteobacterial *amo*A OTUs from Gulf of Mexico and New England marshes. The relative abundance of sequences in each OTU from each region is indicated by the pie charts (blue—GoM, orange—NE). Bootstrap values > 50 are indicated on each branch. The scale bar presents number of substitutions per site.

A total of 740 betaproteobacterial *amo*A sequences were included in the analysis (331 from GoM marshes, 559 from NE marshes), representing three marshes from each region (no sequences from CO or LUM marshes) (Fig. 7). Twenty-six OTUs were detected, including six singletons. Only five OTUs were shared between the two regions, and no OTU was found in all six marshes. NE marshes had a total of 17 OTUs, with 66% of sequences related to *Nitrosospira*. GoM marshes had a total of 16 OTUs, with 77% of sequences related to *Nitrosomonas*. Eight OTUs, not including singletons, were unique to NE marshes, while seven OTUs were unique to GoM marshes. OTUs 4 and 8 were diagnostic for NE marshes, while no OTUs were diagnostic of GoM marshes. Five of the OTUs were unique to a particular marsh, not counting doubletons or singletons. Most OTUs were represented by a single TRF.

Similar to the analyses we conducted with abundance data to investigate possible differences due to depths, analysis of TRFLP data from 0 to 5 cm samples in the GoM marshes were compared to a subset of samples from 0 to 4 cm in NE marshes. Community patterns for both AOA and AOB were virtually identical to the results from the data set containing only 0–2 cm samples from NE (data not shown).

### Comammox clade A diversity

We obtained a small set of comammox clade A *amo*A sequences (98 total) from GoM (71 sequences) and NE (27 sequences) samples. Obtaining quality sequence reads from the NE marshes proved to be more difficult than from the GoM marshes, likely due to the low abundance of comammox in the samples. We found a total of seven OTUs, with four OTUs represented by singletons. OTU1 comprised 64% of the sequences, and was dominated by sequences from GoM samples, while OTU2 was dominated by sequences from NE, and OTU3 was exclusively comprised of sequences from GoM. Most of the OTUs were most closely related to *Candidatus* Nitrospira nitrificans. OTU3 clustered with *Nitrospira inopinata* (Fig. S5).

### AOA and AOB diversity

AOB diversity (both richness and Simpson’s index) was significantly higher in NE marshes, when measured by both TRFLP and sequence data (Table 3). Diversity patterns for AOA between the two regions, however, were less clear. Based on TRFLP data, AOA richness was greater in NE, but Simpson’s was lower in NE compared to GoM, and no differences were detected for either metric using AOA sequence data. Due to the low number of sequences obtained for comammox, we did not compute diversity metrics.

**Table 3:** Average (SE) diversity indices for AOA and AOB in GoM and NE marshes.

Group	Region	Diversity based on TRFLP		Diversity based on sequences	
		Richness^1^	Simpson’s	Richness^2^	Simpson’s
AOA	GoM	5.16 (0.06)^a^	0.62 (0.005)^a^	13.0 (2.6)	0.59 (0.06)
	NE	5.61 (0.14)^b^	0.53 (0.009)^b^	14.0 (3.7)	0.73 (0.02)
AOB	GoM	4.27 (0.10)^a^	0.49 (0.01)^a^	7.0 (0.6)	0.63 (0.04)^a^
	NE	5.47 (0.14)^b^	0.60 (0.01)^b^	10.3 (3.8)	0.81 (0.05)^b^

Different letters indicate significantly different values (*P* < 0.05) between the two regions.

^1^Number of TRFs.

^2^Number of OTUs.

## Discussion

We conducted a meta-analysis of nitrifier data collected over 16 years from two geographic regions to determine the impact of biogeography on microbial communities and ecosystem processes. Relationships between nitrification rates and nitrifier abundances and communities suggest significantly different N cycling dynamics in GoM and NE marshes that are likely driven by environmental differences. We highlight a potentially important role of comammox in the GoM marshes, and we identify common factors, such as salinity and soil moisture, that may be partly responsible for the observed patterns of activity, abundance, and community composition in the two regions.

We set out to answer several questions about nitrifiers in GoM and NE marshes. The first was: Does the abundance and composition of AOA and AOB differ significantly among geographically distant marshes that differ significantly in nitrification rates? Given the higher rates in GoM marshes, we expected to see higher nitrifier abundances accompanying these rates. Although AOA abundances were significantly higher in GoM, we were surprised by the significantly higher abundances of AOB in NE marshes. One might predict that AOB would be favored in the higher nitrogen-loading environments that have been well-documented in the GoM,^34,35^ since cultured AOB have higher *K*_*m*_ values than AOA,^7^ and some have suggested using ratios of AOA to AOB as an indicator of pollution.^36,37^ However, current culture collections represent only a small fraction of the diversity found in the marshes, making accurate ecophysiological predictions difficult. AOB may also have higher tolerance of harsh winter conditions in NE marshes, compared to AOA. Controlled experimental studies will be important to understand the distribution patterns more fully. Regardless, different patterns of nitrifier abundances suggest that there are different dynamics driving nitrification activity between the two regions.

AOA abundances in both GoM and NE marshes are generally higher than in other studies, although they fall within the reported range of 10^4^–10^9^ copies/gram of sediment. Zhang et al.^38^ also reported abundances of 10^9^ copies g^−1^ in mangroves invaded by *S. alterniflora*. Some of the variability in reported values from the literature may be due to differences in the primers used. We found significant primer bias in the NE samples when we used different primers for archaeal *amo*A genes. When using the commonly-used ArchAmoAF/R or Arch26F/417R, abundances were 2–3 orders of magnitude lower than when we used ArchAmoAQModF with ArchAmoAR. The higher abundances detected with the latter primer set were confirmed by quantification of *Nitrosopumilus* 16S rRNA genes, as reported in Moin et al.^11^, so we think it is unlikely that the higher values are due to mispriming events. Based on analysis of archaeal *amo*A sequences, the primers developed by Park et al.^29^ have several mismatches with some archaeal *amo*A genes. However, we tested samples from GoM with the different primer sets and found no significant difference in abundances,^17^ likely reflecting the significant differences in AOA communities between NE and GoM marshes. Others have also reported significantly different results with different primer pairs for AOA,^39^ and significant primer bias of AOA primers was recently documented with plasmid DNAs^40^ and with sediments.^41^

Similar to abundance patterns of AOA and AOB between regions, AOA and AOB communities also differed significantly between the two regions. Differences in the relative abundance of nearly all AOA TRFs between the regions could potentially indicate significant growth differences that would help explain differences in rates. However, we do not have cultured representatives of these AOA, so we can only speculate that some of the more abundant AOA in the GoM may have higher growth rates compared to the abundant AOA in NE marshes.

Interestingly, AOA communities in both GoM and NE marshes were dominated by sequences related to *N. maritimus*, suggesting that there may be core AOA populations shared among all the marshes. This is somewhat surprising since laboratory studies have reported *N. maritimus* as an oligotrophic ammonia oxidizer, with high affinity for ammonium.^7^ Our study is based on the *amo*A gene, so we cannot make definitive identifications without having 16S rRNA sequences as well. However, in the BI salt marsh, previous research showed dominance of both 16S rRNA and *amo*A genes closely related to *N. maritimu*s,^11,19^ suggesting that there may be related strains that are adapted to higher nutrient conditions typically found in marsh sediments.

AOB communities also differed significantly between regions. Based on DNA sequences, GoM marshes were dominated more by *Nitrosomonas*-related AOB, compared to NE marshes that were dominated by *Nitrosospira*-related AOB. Cultured *Nitrosomonas* have higher growth rates compared to cultured *Nitrosospira,*^42^ and these differences may also contribute to the significant differences in nitrification rates between the regions.

Unlike AOA, there were no common AOB found in all of the marshes, suggesting that local marsh conditions may be more important in the selection and maintenance of dominant AOB in each marsh, and perhaps more endemism among AOB compared to AOA. In a global study of AOB based on 16S rRNA analysis in 12 different salt marshes, Martiny et al.^3^ reported that diversity of Nitrosomonadales was most strongly correlated with environment, rather than geographic distance, across continental scales. They also reported that the relatively common AOB taxa appear to be globally distributed, but could not rule out the possibility that endemism occurs. Our analysis based on *amo*A genes provides a similar pattern to that found with 16S rRNA genes reported by Martiny et al.^3^, in that we found some taxa shared between the two regions, although no taxon was found to be ubiquitous, and some taxa were found only in a single marsh.

Diversity patterns also differed between the regions. Greater diversity of AOB in NE marshes relative to GoM marshes parallels their greater abundance and greater percentage of total bacteria. AOA diversity follows a similar pattern, although not as strong, with the exception of Simpson’s index based on TRFLP data. Having higher nitrifier diversity in NE marshes is opposite of what would be predicted based on latitudinal gradient studies of most animals and plants,^43^ and bacterioplankton.^44^ However, many groups of microorganisms may not follow the patterns observed in microorganisms. A continental-scale study of soil bacteria revealed little impact of latitude on bacterial diversity.^45^ And, more recently, Hendershot et al.^46^ explored drivers of microbial diversity in belowground habitats, and found no universal trend in patterns of diversity related to latitude.

Not only were there differences in AOA and AOB abundances, we also found significantly different abundances of total bacteria. We think higher abundances of total bacteria in GoM marshes is likely due to higher organic carbon content of sediments compared to NE marshes. Although we have organic C data from GoM samples, we have organic C data only from a subset of PIE samples in NE marshes, so we chose not to include these data here. However, analyzing the data we had, there was a significant correlation between % C and bacterial 16S rRNA abundance (*r* = 0.35, *P* < 0.0001). This relationship is similar to what others have also reported in estuarine environments.^47,48^

Another consequence of higher total bacterial abundance in GoM marshes is significantly different ratios of nitrifiers to bacteria. The significantly higher ratios of nitrifiers to bacterial 16S rRNA genes in NE marshes compared to GoM marshes suggest that nitrifiers may be more important community members in NE marshes. Given the differences in N loading between the systems, one could argue that N may be a more precious commodity in NE marshes, thus supporting a larger proportion of the community devoted to nitrification. Nitrogen limitation has been well-documented in temperate estuarine systems,^49^ but the N limitation paradigm may shift to phosphorus limitation in more sub-tropical or tropical systems, such as the GoM.^50,51^ If nitrogen limitation is more severe in NE marshes, there may be higher proportions of the microbial community devoted to nitrogen processes.

The second question we set out to answer was: What are the factors that drive spatial variability of AOA and AOB? In order to answer this question, we must also consider the significant differences in the nitrification rates between the regions. The nitrification rates in GoM marshes are generally higher than those reported in other salt marshes,^6^ although they are comparable to rates reported in unvegetated estuarine sediments in Denmark.^52^ We measured rates from only two NE marshes (BI and PIE), but rates from GSM have been previously reported,^53^ and ranged from below detection to 307 nmol g^−1^ d^−1^, comparable to the highest rates reported in BI and PIE of 268 and 240 nmol g^−1^ d^−1^, respectively, suggesting that the differences between GoM and NE marshes are likely region specific.

Factors that drive the high rates in the GoM also likely drive much of the variation in abundance and community composition between the regions. High rates in the GoM may partly be a reflection of environmental variation between the two regions. For example, warmer temperatures in the GoM relative to NE marshes, may be important in regulating activity. Nitrifiers may be more efficient in the GoM due to higher temperatures year-round compared to NE, where colder temperatures during winters may stunt microbial activity for many months. The Metabolic Theory of Ecology predicts that organisms living at higher temperatures tend to have higher metabolic rates than organisms living in colder temperatures,^54^ and could explain higher rates in GoM marshes.

Temperature may also play a role in which nitrifiers contribute to nitrification at different temperatures. Taylor et al.^55^ found that AOA had maximal nitrification rates between 30 and 37 °C, while AOB had maximal rates at 16 and 23 °C in agricultural soils, suggesting that AOA and AOB may contribute differentially to nitrification along a temperature gradient. Similarly, Ouyang et al.^56^ found that AOA in soils had an optimal temperature that was 10 °C higher than AOB. Mukhtar et al.^57^ reported that soils with higher AOA to AOB ratios had higher temperature optima for nitrification. With higher AOA to AOB ratios in the GoM compared to NE marshes, we would predict higher rates at the warmer temperatures in the GoM.

Fierer et al.^58^ found a strong correlation between temperature and soil AOB community composition, suggesting that temperature selects for specific lineages. In our study, it is likely that temperature, along with salinity, is a strong driver of the different ratios of *Nitrosospira* and *Nitrosomonas* lineages in the two regions. Others have also found temperature to be an important factor in regulating AOA and AOB diversity.^59–61^ Average water temperatures in the GoM are 8–12 °C warmer in summer months than temperatures in NE waters, so temperature could be an important factor in regulating differences in diversity and abundance. However, given that mean nitrification rates in GoM and NE varied by more than an order of magnitude, temperature alone is likely not the only factor. Additionally, all our potential rate measurements were done at similar temperatures, suggesting that the differences in rates reflect differences in the community.

Differences in salinity between the two regions may also contribute to differences in rates, since increases in salinity are known to reduce available ammonium in sediments,^62^ and salinity has been identified in other studies as an important factor for nitrifiers (reviewed in ref. ^2^) Our results suggest a strong impact of salinity on rates, as well as nitrifier abundance, with higher rates and abundances, generally, at intermediate salinities. Maximum rates in NE marshes were found at salinities between 10.1 and 20 psu, which generally agrees with a previous study of salinity vs rates in PIE where they found highest rates at 10 psu,^24^ regardless of in situ salinity. Maximum rates at higher salinities (20.1–30 psu) in GoM marshes are somewhat contrary to expectations, since in situ salinities were significantly higher in NE marshes. This seeming contradiction suggests that salinity may not be the overriding factor in determining nitrification rates in the marshes, and that other factors such as nitrogen, oxygen, or temperature may drive rates up, even at salinities less than optimal for the organisms. Marton et al.^6^ found strong correlations between nitrification rates and organic C and total N in GoM marshes, while Schutte et al.^25^ found nitrification correlated with belowground plant biomass, suggesting potential competition for N as an important factor in GoM marshes.

Other factors that we did not measure, such as oxygen and bioturbation, may also be important in the differences between the regions. Enhanced nitrification rates have been reported in marsh sediments with high macrofaunal burrows,^63^ and Beman et al.^64^ found active nitrification as deep as 10 cm, attributing this activity to bioturbation activity at these depths. We have no evidence to suggest that oxygen differs significantly between regions, but future studies should include oxygen measurements to better understand how this may impact nitrifiers in these marshes.

Thirdly, we set out to answer: Are there common factors that regulate AOA and AOB abundance and diversity in salt marshes? Factors regulating AOA abundance were generally similar in both regions, based on correlations of abundance and sediment properties, with soil moisture and pH both significantly correlated with AOA abundance. Others have reported pH to be a major factor for AOA communities in soils,^65,66^ and recent studies of soil drying and rewetting suggest soil moisture is an important factor.^67–69^ The general lack of correlation of sediment properties and AOB abundance in both regions (except for soil moisture in NE) suggests that AOA and AOB abundance are differentially regulated, with AOB abundance responding to factors not measured in this study.

As we were conducting this study of AOA and AOB, the discovery of the complete nitrifier, comammox, was published, so we added a fourth question: Are comammox bacteria potentially an important player in these marshes? We detected comammox in both regions, with significantly higher abundances in GoM marshes, often outnumbering AOB. Significantly higher comammox abundances in the GoM marshes may also contribute to the higher rates.

Studies of the growth kinetics of the cultured comammox *Nitrospira inopinata* indicate slow growth, but high growth yields relative to the canonical ammonia oxidizers.^8^ However, similar to AOA and AOB, there may be a broad range of growth kinetics among comammox bacteria, so it is difficult to extrapolate to the comammox found in GoM and NE marshes. How comammox respond to other variables such as salinity and temperature has yet to be determined. Some have found that increased temperature may favor comammox,^70,71^ while others have found them to thrive under low temperatures.^72^

This is one of the first reports of comammox in salt marshes (see,^73^) and we think it is noteworthy that they are more abundant, in some cases, than AOB. Xia et al.^74^ reported comammox clade A bacteria in coastal waters and sediments, sometimes outnumbering AOB, and comprising up to 35% of aerobic ammonia oxidizers in a metagenomic data set. And, Yu et al.^75^ reported high diversity of comammox clade A in tidal sediment enrichment cultures. Zhao et al.^76^ also found higher comammox compared to AOB in some river sediments, but abundances were lower, and sometimes undetectable, in intertidal sediments. Distribution in more open ocean waters remains uncertain, although in a metagenomic survey, comammox genes were barely detectable.^74^ We found no reports of comammox clade B genes in marine systems. Further studies are necessary to more fully characterize the distribution and abundance of comammox in coastal and ocean systems, and the factors that regulate them.

Some of the initial questions we set out to answer were whether nitrifier abundance and community composition differed between marshes with significantly different nitrification rates, and can we identify factors that drive the differences? The answer to the first question is an unequivocal “yes,” but the answer to the second part is more complex. Although salinity appears to be important in regulating patterns of nitrifier activity, abundance, and community composition, and there were some common factors correlated with AOA abundance in both regions, our data suggest that there is a complex interplay of environmental factors that work together to produce the observed patterns. Thus, it is difficult to provide a satisfyingly simple answer to the questions of what factors drive spatial variability, and what are the common factors that regulate marsh nitrifiers. We do, however, feel more confident in asserting that comammox is likely an important player in GoM marshes, and warrants further study to more fully understand the dynamics among AOA, AOB, and comammox in these coastal systems.

In conclusion, our data suggest there is strong biogeographical provincialism that encompasses not only taxonomic differences, but differences in ecosystem function as well. Our work suggests nitrogen is processed by different groups in different geographic regions, with AOA and comammox being more important in the GoM, while AOB appear to be more important in NE marshes. Who carries out nitrification in these systems can be important in understanding how ecosystem function may be impacted since the different groups of nitrifiers respond to environmental perturbations differently. They also have different physiological and metabolic characteristics that would be expected to play a role in the efficiency of ammonia oxidation. Our findings also suggest that we have far more work to do in understanding nitrification in complex sediment systems. The new discoveries in nitrogen transformations over the last 20 years has led to major shifts in the paradigm, such that if we had conducted this meta-analysis just 15 years ago, before the discovery of AOA or comammox, our story would be significantly different. Our study also highlights the importance of long-term data sets to provide researchers the ability to make these comparisons between systems and groups of organisms.

## Supplementary information

Supplemental Material.
