# Supplementary material for: Biogeography of ammonia oxidizers in New England and Gulf of Mexico salt marshes and the potential importance of comammox
Source: ISME Commun. 2021 Mar 29;1:9. doi: 10.1038/s43705-021-00008-0 (PMC9723745; doi:10.1038/s43705-021-00008-0)
Supplement: Supplementary file 1 — Supplemental Material. [file 43705_2021_8_MOESM1_ESM.docx]

Supplemental file: Biogeography of ammonia oxidizers in New England and Gulf of Mexico salt marshes and the potential importance of comammox

Table S1. Number of samples and sequences used in the study from each region and marsh and the month and year they were collected.

| Region | Marsh | Date | QPCR | | | | Rates | Salinity | NH_4_^+^ | %water | TRFLP | | *amo*A sequences | | |
| --- | --- | --- | --- | --- | --- | --- | --- | --- | --- | --- | --- | --- | --- | --- | --- |
|  |  |  | AOA | AOB | Com | 16S |  |  |  |  | AOA | AOB | AOA | AOB | Com |
| GoM | TB | 5/2012 | 16 | 16 |  | 16 | 16 |  |  | 16 | 16 |  |  |  |  |
|  |  | 7/2012 | 16 | 16 | 16 | 16 | 15 |  |  | 16 | 16 | 16 | 142 | 83 |  |
|  |  | 8/2012 | 15 | 15 |  | 15 | 15 |  |  | 16 | 16 | 3 |  |  |  |
|  |  | 9/2012 | 16 | 16 |  | 16 | 15 |  |  | 16 | 16 | 13 |  |  |  |
|  |  | 3/2013 | 16 | 16 |  |  |  |  |  | 16 |  | 16 |  |  |  |
|  |  | 5/2013 | 16 | 16 |  |  | 16 |  |  | 16 |  | 9 |  |  |  |
|  |  | 7/2013 | 20 | 20 | 20 |  | 16 |  |  | 16 | 16 | 16 |  |  |  |
|  |  | 9/2013 | 16 | 16 |  |  | 16 |  |  | 16 | 16 | 15 |  |  |  |
|  |  | 4/2014 | 16 | 16 |  |  |  |  |  | 16 |  |  |  |  |  |
|  |  | 5/2014 |  |  |  |  |  |  |  | 16 | 16 | 16 |  |  |  |
|  |  | 7/2014 | 16 | 16 | 16 | 16 | 16 | 14 | 15 | 16 | 16 | 15 |  |  |  |
|  |  | 9/2014 |  |  |  |  |  |  |  | 15 | 15 | 15 |  |  |  |
|  |  | 7/2015 | 36 | 36 | 36 | 16 | 16 | 15 | 15 | 16 | 31 | 16 |  |  |  |
|  |  | 7/2016 | 16 | 16 | 16 |  | 16 | 15 |  | 16 | 15 | 16 |  | 40 | 17 |
|  | WB | 7/2012 | 20 | 20 | 20 | 20 | 20 |  |  | 20 | 20 | 20 | 131 | 84 |  |
|  |  | 9/2012 |  | 20 |  |  | 20 |  |  | 20 | 20 | 19 |  |  |  |
|  |  | 5/2013 | 8 | 8 |  |  | 8 |  |  | 8 |  | 8 |  |  |  |
|  |  | 7/2013 | 15 | 15 | 15 | 15 | 16 |  |  | 16 | 16 | 16 |  |  |  |
|  |  | 9/2013 | 16 | 16 |  |  | 16 |  |  | 16 | 16 | 15 |  |  |  |
|  |  | 7/2014 | 16 | 16 | 16 | 16 | 16 | 16 | 10 | 16 | 16 | 16 |  |  |  |
|  |  | 7/2015 | 28 | 28 | 28 | 15 | 15 | 16 | 13 | 16 | 27 | 16 |  |  |  |
|  |  | 7/2016 | 16 | 16 | 16 |  | 16 | 16 |  | 16 | 16 | 16 | 22 | 64 | 28 |
|  | EB | 7/2012 | 16 | 13 | 8 | 16 | 14 |  |  | 16 | 15 | 15 | 155 | 32 |  |
|  |  | 9/2012 | 16 | 16 |  |  | 15 |  |  | 16 | 15 | 12 |  |  |  |
|  |  | 5/2013 | 16 | 16 |  |  | 16 |  |  | 16 |  |  |  |  |  |
|  |  | 7/2013 | 16 | 16 | 16 | 16 | 16 |  |  | 16 | 16 | 16 |  |  |  |
|  |  | 9/2013 | 15 | 16 |  |  | 16 |  |  | 16 | 16 | 12 |  |  |  |
|  |  | 7/2014 | 16 | 16 | 16 | 16 | 16 | 16 | 13 | 16 | 16 | 13 |  |  |  |
|  |  | 7/2015 | 28 | 28 | 28 | 16 | 16 | 16 | 15 | 16 | 27 | 15 |  |  |  |
|  |  | 7/2016 | 16 | 16 | 16 |  | 16 | 16 |  | 16 | 16 | 16 | 66 | 28 | 26 |
|  | LUM | 7/2015 | 54 | 54 | 26 | 54 | 54 | 54 |  | 54 | 53 | 20 |  |  |  |
|  |  | GoM total | **527** | **545** | **309** | **279** | **463** | **194** | **81** | **533** | **494** | **411** | **516** | **331** | **71** |
| NE | BI | 10/2005 | 16 | 16 | 16 | 15 |  | 10 |  | 16 | 13 | 16 |  |  |  |
|  |  | 3/2006 | 9 | 9 |  |  |  | 6 |  |  |  |  |  |  |  |
|  |  | 4/2006 | 29 | 25 | 24 |  | 6 | 0 |  | 30 | 26 | 23 |  |  | 7 |
|  |  | 6/2006 | 7 | 8 | 13 | 17 | 8 | 6 |  | 22 | 22 | 12 |  |  |  |
|  |  | 7/2006 | 24 | 23 | 27 | 18 | 9 | 30 | 30 | 30 | 27 | 28 | 121 | 233 |  |
|  |  | 10/2006 | 8 | 9 |  | 3 | 9 | 9 |  | 9 |  | 17 |  |  |  |
|  |  | 6/2014 | 12 | 16 | 16 | 12 |  |  |  | 16 | 24 | 14 |  |  |  |
|  |  | 7/2016 | 8 | 9 |  | 8 |  | 7 |  | 9 | 14 | 9 |  |  |  |
|  |  | 6/2017 | 9 | 12 | 11 | 9 |  | 12 |  | 12 | 15 | 12 |  |  |  |
|  | CO | 7/2006 | 8 | 6 | 9 | 6 |  | 9 |  | 9 | 9 | 8 |  |  |  |
|  |  | 10/2006 | 6 | 6 | 6 |  |  | 6 |  | 6 |  | 5 |  |  |  |
|  | PIE | 4/2001 | 26 | 22 | 24 | 24 | 26 | 22 | 6 | 26 | 4 | 3 | 225 | 15 | 20 |
|  |  | 8/2001 | 29 | 23 | 26 | 24 | 29 | 23 | 6 | 27 | 8 | 2 | 92 |  |  |
|  |  | 4/2002 | 6 | 6 | 6 | 6 | 6 | 6 | 2 | 6 | 2 |  | 36 | 14 |  |
|  |  | 9/2002 | 8 | 7 | 8 | 9 | 9 | 9 | 4 | 9 | 1 |  | 8 |  |  |
|  |  | 4/2003 | 3 | 3 |  |  | 3 | 3 | 1 | 3 |  |  | 43 | 15 |  |
|  |  | 9/2003 | 9 | 9 | 9 | 9 | 9 | 9 | 3 | 9 | 2 |  |  |  |  |
|  | GSM | 7/2009 | 16 | 16 | 16 | 16 |  | 16 | 16 | 16 | 16 | 16 | 78 | 282 |  |
|  |  | NE total | **233** | **225** | **211** | **176** | **114** | **190** | **68** | **255** | **183** | **165** | **603** | **559** | **27** |
|  |  | Total | **760** | **770** | **520** | **455** | **577** | **377** | **149** | **788** | **677** | **576** | **1119** | **890** | **98** |

Table S2. Primers and conditions used in QPCR assays of target genes.

| Target Gene | Primers | Conditions | Reference(s) |
| --- | --- | --- | --- |
| Archaeal *amo*A | ArchAmoAQModF/Arch amoAR | 95°C for 15 s, 54°C 20 s, 72°C 45 s | (Francis *et al.*, 2005; Moin *et al.*, 2009) |
|  | Arch26F/417R | 95°C for 15 s, 56°C 20 s, 72°C 45 s | (Park *et al.*, 2008) |
| Bacterial 16S rRNA | GM3/338R | 95°C for 15 s, 55°C 20 s, 72°C 45 s | (Amann *et al.*, 1990; Lane, 1991) |
| Betaproteobacterial *amo*A | amoA1F/amoA2R-TC | 95°C for 15 s, 57°C 20 s, 72°C 45 s, 83°C 10 s (read fluorescence) | (Rotthauwe *et al.*, 1997; Nicolaisen and Ramsing, 2002) |
| comammox *amo*A | comaA-244f_d/comaA-659r_c and comaA-659r_d | 95°C for 30 s, 59°C 45 s, 72°C 45 s | (Pjevac *et al.*, 2017) |


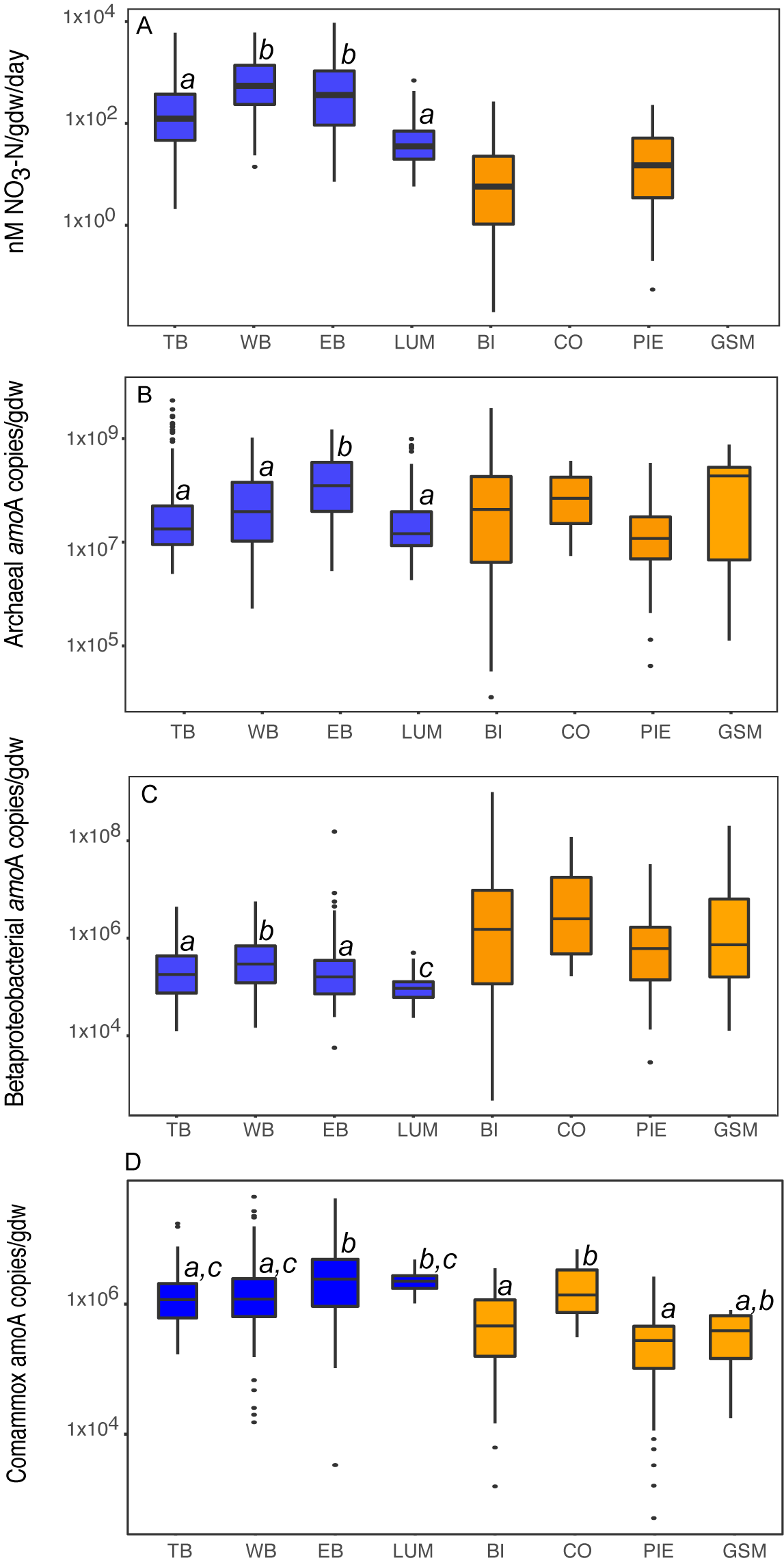


Figure S1. Potential nitrification rates (panel A) and abundance of archaeal *amo*A (panel B), betaproteobacterial *amo*A (panel C), and comammox clade A *amo*A (panel D) in Gulf of Mexico (blue) and New England (orange) marshes. No rates were measured in Cottrell (CO) or Sippewissett (GSM) marshes. Different letters above the boxes indicate significantly different values among marshes within each region.


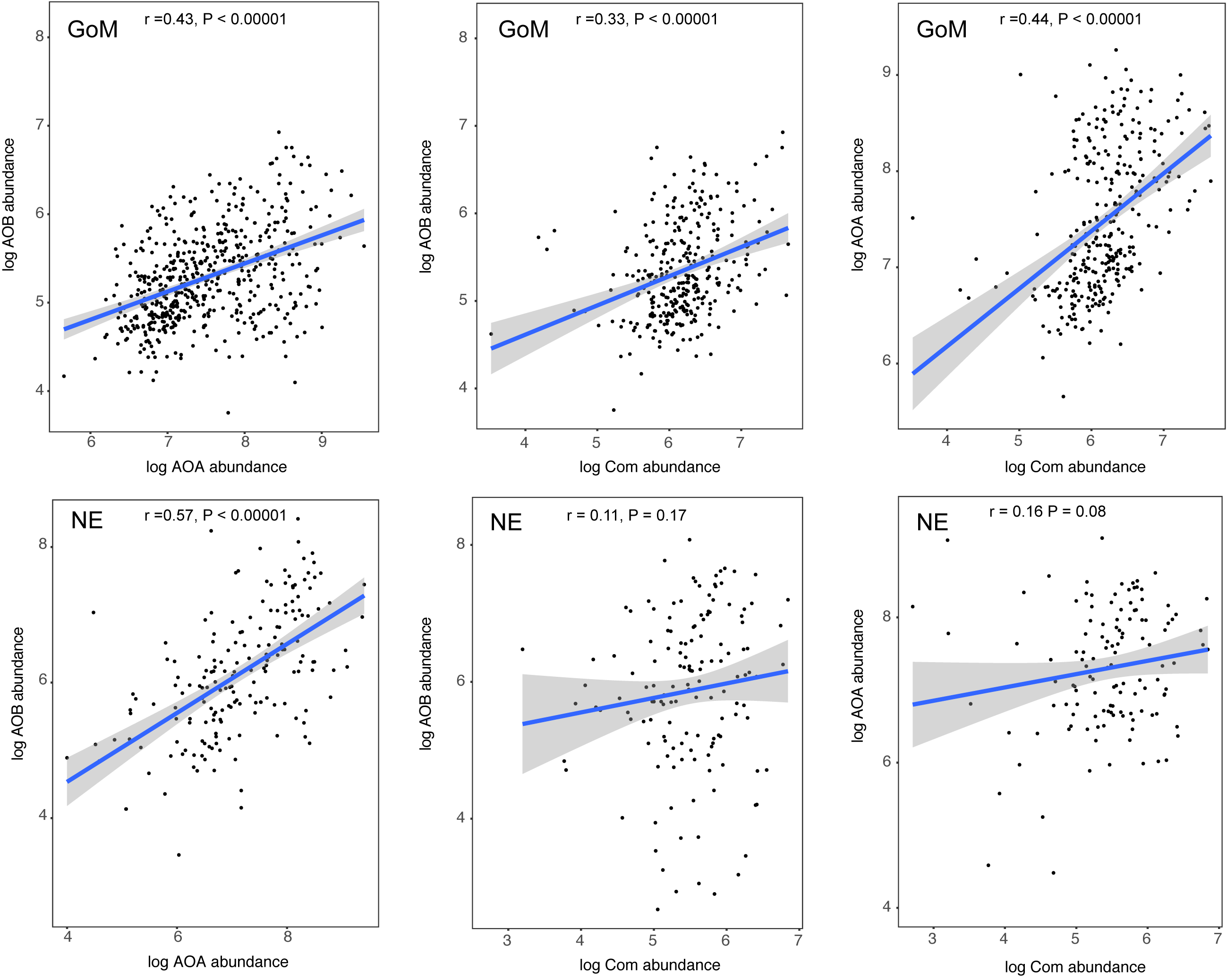


Figure S2. Relationships between AOA, AOB, and comammox abundances. Pearson's correlation coefficients and the P values are shown in each panel.


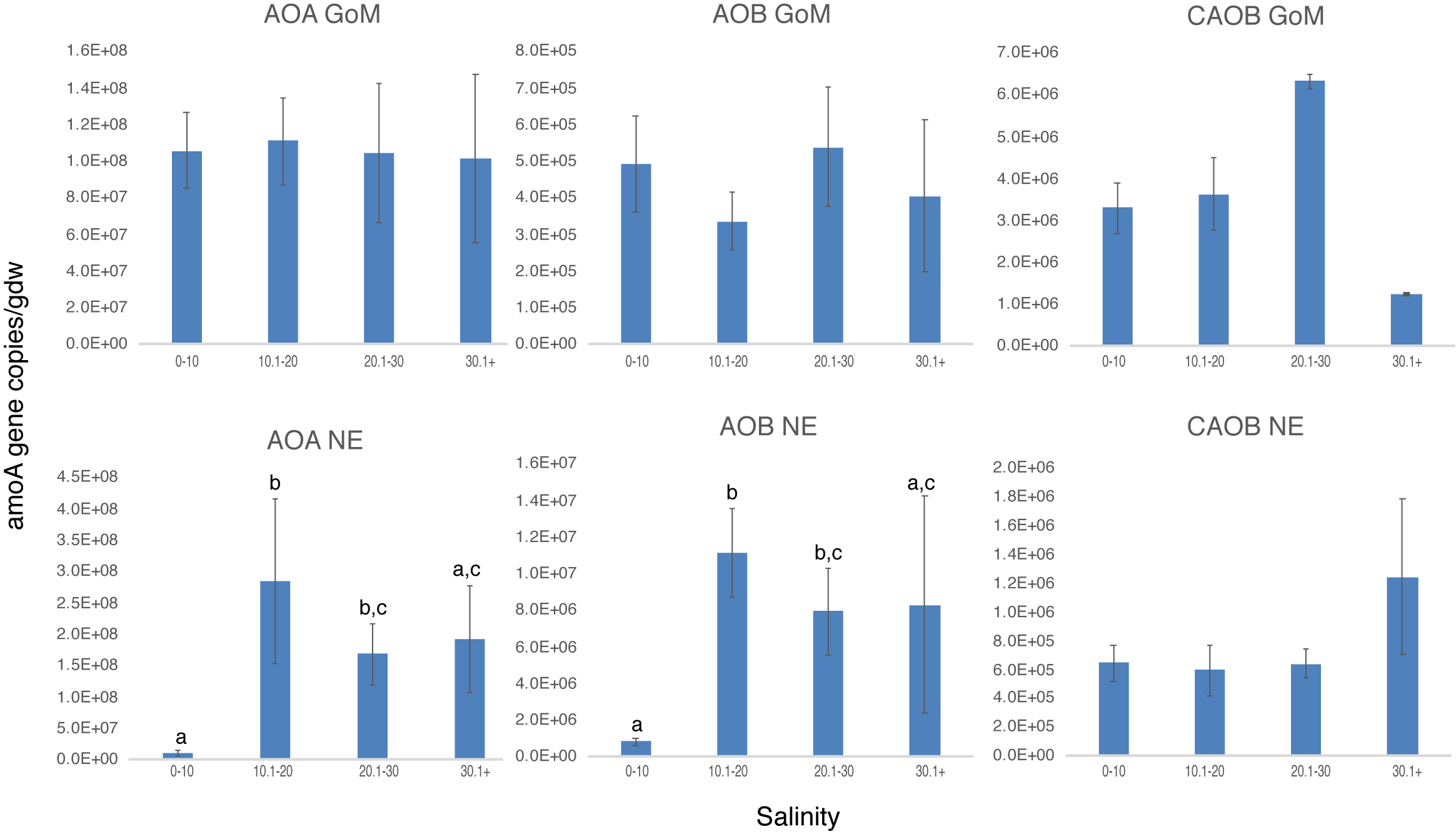


Figure S3. Mean (± SE) abundance of *amo*A genes for AOA (left), AOB (center), and Comammox (right) at different salinities in GoM (top panels) and NE (bottom panels) marshes. Significantly different abundances among salinity categories are indicated by different letters above the bars.


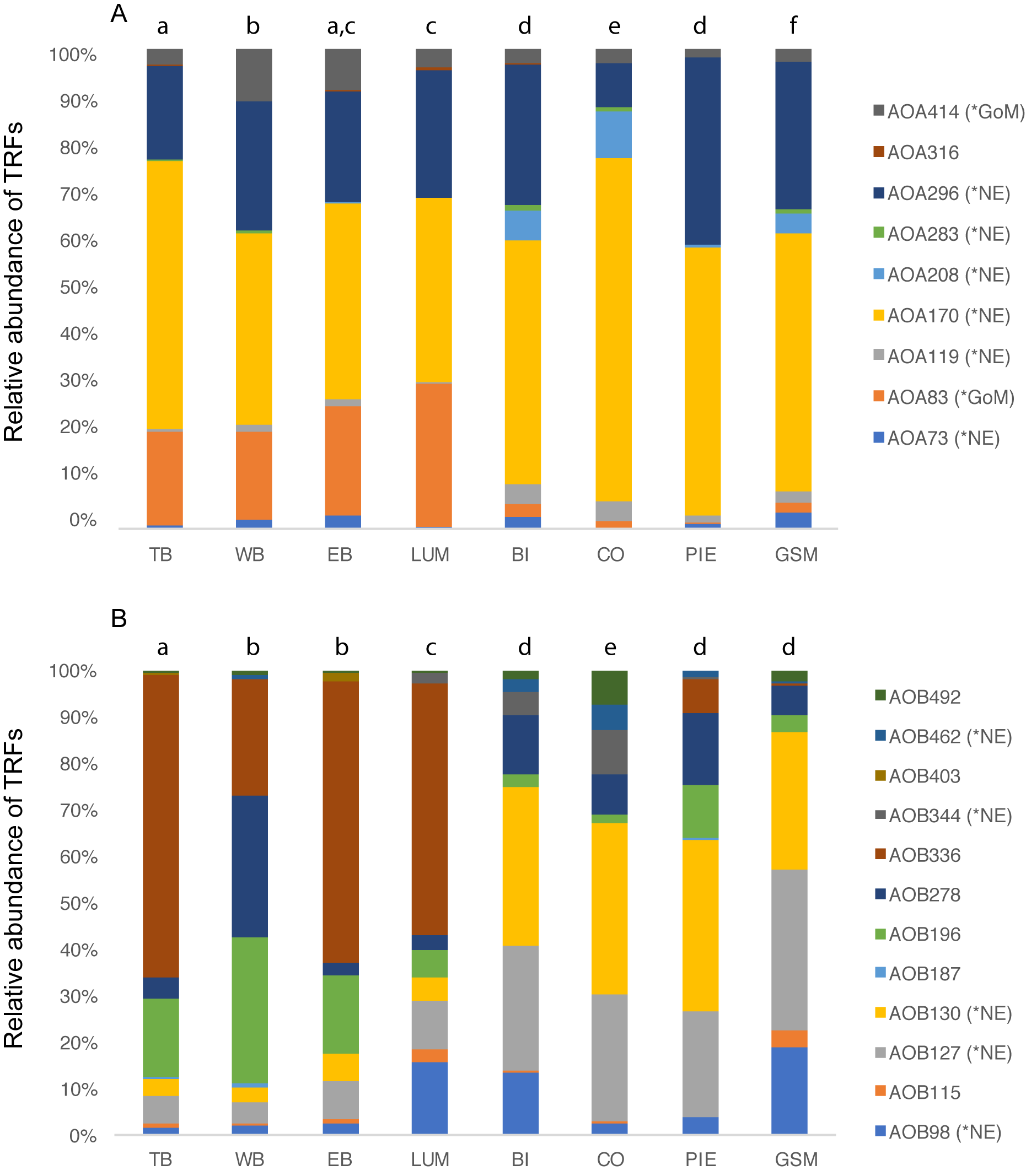


Figure S4. Relative abundance of terminal restriction fragments (TRF) for archaeal amoA genes (panel A) and betaproteobacterial amoA genes (panel B) from Gulf of Mexico (TB, WB, EB, LUM) and New England marshes (BI, CO, PIE, GSM). TRFs are identified by their size in base pairs (shown in the legend on the right), and TRFs that were significantly more abundant in one region are indicated parenthetically in the legend. Communities that were significantly different (by MRPP analysis) are indicated by different letters above the bars.

Figure S5. Phylogenetic relationships among comammox clade A *amo*A genes from Gulf of Mexico (blue circles) and New England (orange circles) marshes. Sequences shown represent the 7 OTUs detected in the data set and number of sequences in each OTU is shown parenthetically.

References

Amann, R.I., Krumholz, L., and Stahl, D.A. (1990) Fluorescent-oligonuleotide probing of whole cells for determinative, phylogenetic, and envrionmental studies in microbiology. *J. Bacteriol.* **172**: 762–770.

Francis, C.A., Roberts, K.J., Beman, J.M., Santoro, A.E., and Oakley, B.B. (2005) Ubiquity and diversity of ammonia-oxidizing archaea in water columns and sediments of the ocean. *Proc. Natl. Acad. Sci. U. S. A.* **102**: 14683–14688.

Lane, D.J. (1991) 16S/23S rRNA sequencing. In, Stackebrandt,E. and Goodfellow,M. (eds), *Nuleic acid techniques in bacterial systematics*. Wiley, pp. 115–175.

Moin, N.S., Nelson, K.A., Bush, A., and Bernhard, A.E. (2009) Distribution and Diversity of Archaeal and Bacterial Ammonia-Oxidizers in Salt Marsh Sediment. *Appl. Environ. Microbiol.* **75**: 7461–7468.

Nicolaisen, M.H. and Ramsing, N.B. (2002) Denaturing gradient gel electrophoresis (DGGE) approached to study the diversity of ammonia-oxidizing bacteria. *J. Microbiol. Methods* **50**: 189–203.

Park, S.J., Park, B.J., and Rhee, S.K. (2008) Comparative analysis of archaeal 16S rRNA and amoA genes to estimate the abundance and diversity of ammonia-oxidizing archaea in marine sediments. *Extremophiles* 1–11.

Pjevac, P., Schauberger, C., Poghosyan, L., Herbold, C.W., van Kessel, M.A.H.J., Daebeler, A., et al. (2017) AmoA-targeted polymerase chain reaction primers for the specific detection and quantification of comammox Nitrospira in the environment. *Front. Microbiol.* **8**: doi: 10.3389/fmicb.2017.01508.

Rotthauwe, J.H., Witzel, K.P., and Liesack, W. (1997) The ammonia monooxygenase structural gene amoA as a functional marker: Molecular fine-scale analysis of natural ammonia-oxidizing populations. *Appl. Environ. Microbiol.* **63**: 4704–4712.
